# Does brief intervention work for heavy episodic drinking? A comparison of emergency department patients in two cultures

**DOI:** 10.1186/1940-0640-10-S2-O11

**Published:** 2015-09-24

**Authors:** Cheryl J Cherpitel, Yu Ye, Jacek Moskalewicz, Robert Woolard

**Affiliations:** 1 Alcohol Research Group, Emeryville, CA, USA; 2 Institute of Psychiatry and Neurology, Warsaw, Poland; 3 Texas Tech University Health Science Center, El Paso, TX, USA

## Background

Little has been reported on the efficacy of brief intervention (BI) among heavy episodic drinkers, although this drinking style is known to be especially harmful in relation to negative consequences including alcohol-related injuries.

The objective is the comparative efficacy of BI is analyzed in two similar randomized controlled clinical trials of emergency department (ED) patients in two different cultures, both of which exhibit similar drinking styles of heavy episodic drinking: Poland and Mexican-Americans in the U.S.

## Material and methods

Improvements in drinking and problem outcomes are analyzed at 3-month and 12-month follow-up, using random effects modeling, among 446 Polish patients and 698 Mexican-American patients, randomized to screened only, assessment, and intervention conditions in each study.

## Results

In Poland significant improvement was observed in all outcome measures for the assessed condition at 3-months compared to baseline, but only in the two problem variables at 12-months, while for the intervention condition, significant improvement was found in all outcome measures at both time periods; however, estimates of the interaction terms were not statistically significant. In the Mexica-American study, while significant improvement in nearly all outcome measures were observed at 3 months and 12 months for both conditions, estimates of the interaction terms suggest that for all drinking variables, but not problem variables, outcomes were significantly improved for the intervention condition over the assessed condition at 12 months, suggesting a 12-month treatment effect.

## Conclusions

Findings here are non-conclusive regarding a treatment effect of BI for heavy episodic drinking in ED patients. Given the mixed findings found for BI in other ED studies, future studies are needed to explore the efficacy of BI in other populations and cultures exhibiting different drinking patterns to help identify what type of drinker would most benefit from BI in the ED setting.

